# Assessing Lateral Interaction in the Synesthetic Visual Brain

**DOI:** 10.3390/vision3010007

**Published:** 2019-02-08

**Authors:** Diana Jimena Arias, Anthony Hosein, Dave Saint-Amour

**Affiliations:** 1 Department of Psychology, Université du Québec à Montréal, Montréal, QC H2X 3P2, Canada; 2 Cognitive Neurosciences Research Center, Université du Québec à Montréal, Montréal, QC H2X 3P2, Canada; 3 Research Center of the Sainte-Justine University Hospital, Montréal, QC H3T 1C5, Canada

**Keywords:** synesthesia, visual evoked potentials, EEG, occipital cortex, lateral interaction

## Abstract

In grapheme-color synesthesia, letters and numbers evoke abnormal colored perceptions. Although the underlying mechanisms are not known, it is largely thought that the synesthetic brain is characterized by atypical connectivity throughout various brain regions, including the visual areas. To study the putative impact of synesthesia on the visual brain, we assessed lateral interactions (i.e., local functional connectivity between neighboring neurons in the visual cortex) by recording steady-state visual evoked potentials (ssVEPs) over the occipital region in color-grapheme synesthetes (*n* = 6) and controls (*n* = 21) using the windmill/dartboard paradigm. Discrete Fourier Transform analysis was conducted to extract the fundamental frequency and the second harmonics of ssVEP responses from contrast-reversing stimuli presented at 4.27 Hz. Lateral interactions were assessed using two amplitude-based indices: Short-range and long-range lateral interactions. Results indicated that synesthetes had a statistically weaker signal coherence of the fundamental frequency component compared to the controls, but no group differences were observed on lateral interaction indices. However, a significant correlation was found between long-range lateral interactions and the type of synesthesia experience (projector versus associator). We conclude that the occipital activity related to lateral interactions in synesthetes does not substantially differ from that observed in controls. Further investigation is needed to understand the impact of synesthesia on visual processing, specifically in relation to subjective experiences of synesthete individuals.

## 1. Introduction

Synesthesia occurs when a stimulus in one sensory modality elicits an additional and abnormal perception in the same or a different sensory modality. One of the most studied types of synesthesia is the grapheme-color type, which consists of letters and numbers evoking colored perceptions (e.g., printed black letters perceived as being colored) [1]. Such synesthetic associations are consistent over time [2,3,4] and difficult to disregard after having been elicited [5,6,7,8]. Synesthetic experiences can, however, differ qualitatively between individuals. For instance, some synesthetes perceive colors as being “outside” of their body, while others perceive them internally, i.e., in the “mind’s eye”. This phenomenological distinction in synesthetic percepts is known as the projector type and the associator type, respectively [8].

Although the underlying mechanisms of synesthesia are still unknown, it is largely thought that the synesthetic brain is characterized by atypical connectivity among various brain regions [9,10,11], including visual areas [12,13,14,15]. Visual processing regions required for color and shape processing, which are located beyond the striate cortex, have been suggested to play an important role in synesthetic grapheme-color perception [13,16,17,18,19]. For instance, a resting-state fMRI study reported that synesthetes shared a global neural network with non-synesthetes (sensory, parietal, and frontal regions). However, synesthetes showed an increased intrinsic internetwork connectivity compared to the controls, in that stronger functional connectivity was observed between the frontoparietal and the lateral visual network, which encompasses many associative regions including the color area V4. Such connectivity was also elicited in the medial visual network, which includes the calcarine gyrus and thus the primary visual cortex (V1) [20]. The involvement of V1 in the circuitry of the synesthetic brain was also corroborated by Sinke et al., [21]. The authors demonstrated that synesthetes had stronger functional connectivity between the parietal lobe and the primary/secondary visual areas compared to control participants. Some evidence suggests that synesthetic hyperconnectivity does not necessarily involve the parietal hub. Indeed, V4 activation during grapheme-color synesthesia has been shown to depend on parietal lobe activation in associator synesthetes, but not in the projector type [22]. In the latter, direct bottom-up cross-activation within the visual cortices was sufficient to account for the synesthetic experience.

The aforementioned studies raised the question of whether atypical connectivity in the primary visual cortex of synesthetes is accompanied by atypical processing within V1 itself. Some evidence from transcranial magnetic stimulation (TMS) and visual evoked potential (VEP) studies have demonstrated the presence of atypical activity in the visual cortex of synesthetes [14,15,23,24,25,26]. Using psychophysical tasks that preferentially target V1 computation, Terhune et al. [27] reported no difference in performance between controls and synesthetes, neither for the tilt-after effect task nor the orientation-specific surround suppression task. The latter task is thought to rely on local lateral interactions, which are dependent on the local connectivity networks.

The current study aimed to directly investigate the integrity of lateral interactions in V1 in grapheme-color synesthesia using steady-state visual evoked potentials (ssVEPs). Lateral interaction processing was assessed with the so-called task-free “windmill/dartboard” paradigm [18], which has been used in different clinical populations [28,29,30,31]. In this paradigm, a central pattern is surrounded by three contiguous rings, which are radially divided into light and dark checkers with only the central and the second rings being dynamic (contrast-reversing). When the central pattern and the second ring are presented and the contrast of the first and third static rings are set to 0%, i.e., turned off, the expected component of the ssVEP at the second harmonic of the frequency of modulation is observed. However, when the first and third static rings are turned on, a perceptual transition occurs between a windmill with a small number of contours and a dartboard with a larger number of contours and edges. As a consequence, the fundamental modulation frequency of the ssVEP is amplified and the second harmonic is attenuated, reflecting the lateral-interaction effects of varying the contrast at the common borders of contiguous static and dynamic segments of the windmill/dartboard stimulus. Neural responses to the windmill/dartboard stimulus are thought to reflect local inhibitory and excitatory activity from neighboring neurons (within a 5 mm region) in the human striate cortex [18,32,33]. Because of the putative atypical connectivity in the primary visual cortex in synesthesia, we hypothesized that the brain responses evoked by the windmill/dartboard paradigm will differ in synesthetes compared to controls, reflecting the altered lateral interaction processing in V1.

## 2. Materials and Methods 

### 2.1. Participants

Six female subjects experiencing grapheme-color synesthesia (aged 21 to 33) and twenty-one female control subjects (aged 21 to 36) were recruited for the purpose of this study. Synesthetic associations were first documented using an interview. The presence of synesthesia was then confirmed using the on-line grapheme-color consistency test of the synesthesia battery [3]. Synesthete participants were assessed twice using this battery, with a minimum lapse of two months between each testing session. A score of 1.0 or lower [3] on a consistency test reflects the presence of synesthetic associations. Scores higher than 1.0, in most cases around 2.0, are ranked as arbitrary associations [3]. As expected, the consistency scores of the synesthete participants ranged from 0.43 to 0.91, all within the synesthetic associations rank. Recruited control participants were included if they related no synesthetic associations.

Participants were included in the study if they had no history of neurological or psychiatric disorder. All participants reported normal or corrected-to-normal vision, which was verified with the Snellen acuity chart and the contrast sensitivity FACT test (Stereo Optical Company Inc., Chicago, IL, USA). All participants consented to participate in the study and received financial compensation. The experimental procedure was approved by the Research Ethics Committee of the Human Sciences Faculty of the Université du Québec à Montréal (FSH-2013-92, 19 November 2014).

### 2.2. Stimuli

**Figure 1:**
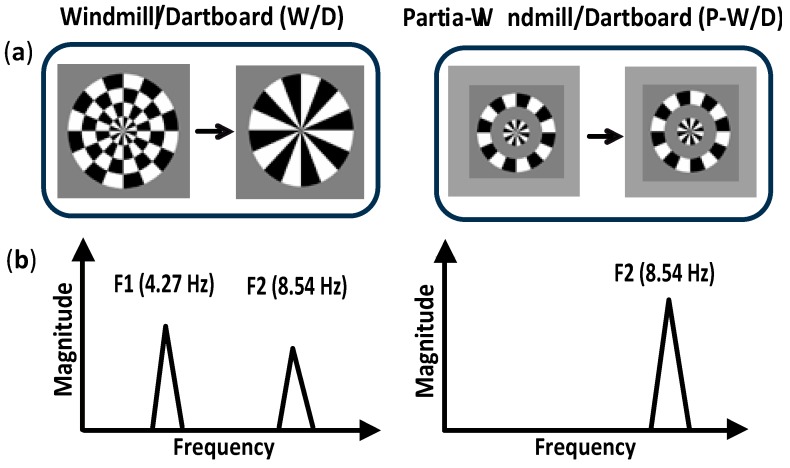
Schematic illustration of the paradigm used to investigate lateral interactions processing (**a**). In the Windmill/Dartboard (W/D) condition (left), the first and third rings (from the center to the outer edge) were static while the center and the second ring were contrast reversing at 4.27 Hz. In the Partial-Windmill/Dartboard (P-W/D) condition (right), only the center and second ring were presented dynamically. (**b**). Illustration of the predicted pattern of steady-state visual evoked potentials (ssVEPs) during the presentation of the W/D (left) and the P-W/D (right) stimuli. The W/D condition is expected to elicit a fundamental frequency or first harmonic (F1) larger than the second harmonic (F2). The second harmonic (F2) in the W/D is attenuated compared to the second harmonic (F2) obtained in the P-W/D. The fundamental frequency (F1) in the P-W/D is absent.

Visual stimuli were composed of a central pattern surrounded by three contiguous rings (each ring being 1° thick) and were presented under two experimental conditions: The Windmill/Dartboard (W/D) and the Partial-Windmill/Dartboard (P-W/D) [18,32] (Figure 1). In the W/D, all radial rings were present at 30% contrast. The first and third rings were static, while the center and the second rings were contrast reversing at a rate of 4.27 Hz, producing changing appearances from a dartboard configuration to a windmill. In the P-W/D, only the two contrast-reversing radial rings were presented (the first and the third rings were set at 0% contrast). Specific response patterns were elicited by the W/D and P-W/D and reflected two distinct types of local lateral interactions within the primary visual cortex [18,30]. Modulations of the neural response at the fundamental frequency (4.27 Hz), only detectable in response to the W/D, were associated with short-range lateral interactions whereas an attenuation of the second harmonic in response to the W/D were associated with long-range lateral interactions [33,34]. Short-range lateral interactions represent foveal projections over a cortical hyper-column width, while long-range connections extend beyond the region covered by short-range lateral interactions [18].

Stimuli were presented on a gray background with a mean luminance of 13 cd/m^2^. Each stimulus presentation lasted 60 s and was shown twice in each condition (W/D and P-W/D). Visual stimulation was executed using a PC computer with an Intel core2 dual processor using the Presentation software (Neurobehavioral Systems Inc., CA, USA) and displayed on a ViewPixx EEG monitor (Vpixx technologies Inc., Saint-Bruno, QC, Canada).

### 2.3. Procedure

The procedure took place in a dimly lit and sound-attenuated room. Participants viewed the stimuli on the monitor from a distance of 57 cm, which was standardized using a chin rest. They were instructed to remain as relaxed as possible and to avoid blinking during the trials. Additionally, they were asked to maintain their gaze onto the central fixation dot while the images were presented. Participants pressed the “ENTER” key to pass to the following trial. 

### 2.4. EEG Data Acquisition and Data Analysis

EEG data were acquired using the V-AMP system (Brain Products, Inc., Munich, Germany) with Ag/AgCl electrodes, and recorded at a sampling rate of 1000 Hz with an online band-pass filter from 0.1 to 100 Hz. The active EEG signal was recorded from an electrode placed at Oz, the reference electrode was placed at Fz, and the ground electrode on the forehead. Electrode placement was based on the international 10–20 system and followed the standard practice suggested by the International Society for Clinical Electrophysiology of Vision [35]. Electrode impedances were verified to be below 5 Ω. An offline band-pass filter of 1 Hz high-pass and 50 Hz low-pass was applied to the data for each participant. Data analyses were performed using the Brain Vision Analyser software as well as Matlab 2015a (MathWorks, Inc., Natick, MA, USA). Data from the W/D and P-W/D conditions were segmented in epochs of 6 s, for a total of 20 epochs per condition. A discrete Fourier transform was computed for every epoch to obtain the magnitude and the phase of the frequencies of interest, that is, the fundamental frequency (F1) of 4.27 Hz and the second harmonic (F2) of 8.54 Hz. For each signal of interest (i.e., 4.27 or 8.54 Hz), magnitude values were corrected for noise by subtracting the average magnitude of two neighboring (±0.5 Hz) frequencies [36].

An objective signal detection technique was first applied to the spectral responses for each participant using the magnitude-squared coherence (MSC) [37,38,39], which corresponds to the ratio of signal power to signal + noise power for a given frequency component. Thus, MSC was calculated from the magnitude and phase of the fundamental frequency (4.27 Hz) as well as the second harmonic (8.54 Hz) to estimate the proportion of the signal relating to these frequencies. An MSC value approaching 1 is indicative of a notable signal, while a score approaching 0 indicates a weak signal that cannot clearly be distinguished from the background noise. Due to the fact that MSC estimation is influenced by the sample size (number of EEG epochs), a bias level threshold is determined as 1/sample size, i.e., 1/20 or 0.05 in the present study, and the calculated bias becomes the “zero point” of the MSC scale for the given analysis. Any MSC value falling below this “zero point” is considered to be too noisy to have any meaningful value. On this basis, the data of two control participants were excluded from different analyses due to MSC values below the bias level of 0.05.

Two lateral interaction indices were calculated from the magnitude of the frequencies of interest evoked by the W/D and P-W/D [28,30]. First, the so-called short-range lateral interaction index was computed from the ratio of the magnitude of F1 over the magnitude of F2 in the W/D condition (SR-LI index). This index reflects the normalized magnitude of the fundamental frequency in the W/D condition. Second, a long-range lateral interaction index was calculated from the attenuation of the second harmonic in the W/D condition as follows: Magnitude of F2 in the P-W/D condition over the magnitude of F2 in the W/D condition (LR-LI index). A higher score in both indexes suggests stronger lateral interactions in V1.

Separate statistical analyses were conducted for the MSC, SR-LI index, and the LR-LI index. Given the unequal sample size between groups and the abnormal distribution of variables, group differences were estimated using non-parametric tests for each variable (Mann–Whitney, exact test). For MSC measures, a total of four between-group comparisons were conducted: F1 of the W/D condition, F2 of the W/D condition, F1 of the P-W/D condition, and F2 of the P-W/D condition. The statistical significance α level of <0.05 was therefore adjusted to <0.0125 using the Bonferroni correction (0.05 divided by 4 comparisons). For lateral interaction indices, a total of 2 comparisons (SR-LI index and LR-LI index.) were performed and the statistical significance α level of <0.05 was corrected to <0.025. The raw *p*-values in the results section were therefore tested for statistical significance against the aforementioned corrected α values.

## 3. Results

**Figure 2:**
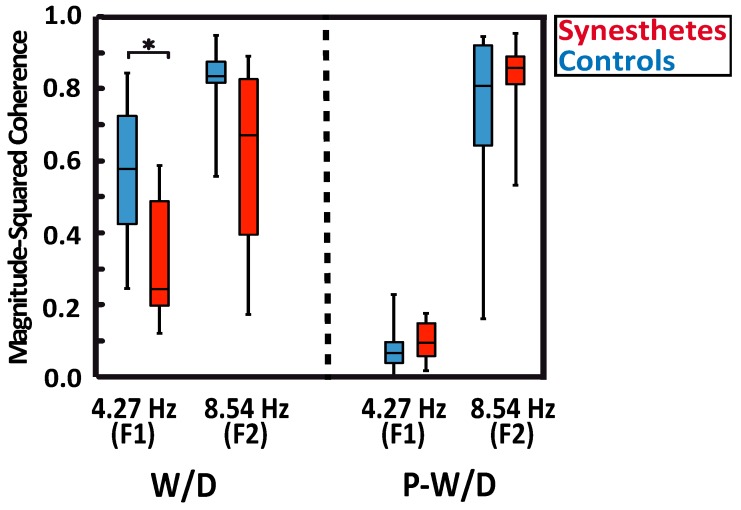
Magnitude-squared coherence (MSC) of ssVEPs responses in synesthetic participants (red), and control participants (blue). The whiskers indicate the minimum and maximum range of the distributions, the top and bottom of the boxes show the first and third quartiles (25th and 75th percentile), and horizontal bars inside the boxes represent the medians.

MSC analyses showed robust responses for all subjects at the fundamental frequency (F1), at the second harmonic (F2) in the W/D condition, and at the second harmonic of the P-W/D condition (Figure 2). As expected, the MSC for the P-W/D at the fundamental frequency was at noise level when compared to the W/D condition. As depicted in the graph (Figure 2), all participants exhibited higher signal coherence at the second harmonic than at the fundamental frequency for both experimental conditions. The only significant difference revealed in this analysis was obtained at the fundamental frequency of the W/D condition (U = 21, *p* = 0.012, *r* = 0.471, exact significance). Thus, the response of synesthetic participants (median = 0.243) at this frequency was significantly weaker than that of the control participants (median = 0.578). Although the pattern remains present for the second harmonic of the W/D, the test did not reach statistical significance (U = 32, *p* = 0.075, *r* = 0.347, exact significance; synesthetes: Median = 0.670 vs. controls: Median = 0.835). No significant group difference was detected at the fundamental frequency in the P-W/D condition (U = 50, *p* = 0.476, *r* = 0.145, exact significance). Thus, for both synesthetes (median = 0.095) and controls (median = 0.066), MSC scores were similar to the noise level (0.05). In addition, no group differences were found in relation to the second harmonic (U = 56, *p* = 0.712, *r* = 0.078, exact significance), such that the synesthete (median = 0.857) and control (median = 0.808) groups showed similar signals at the second harmonic in the P-W/D condition. In sum, MSC analyses suggest that synesthetes exhibited a lower signal coherence and a higher level of noise (i.e., more neural variability) than controls, but only for the fundamental frequency response in the W/D condition.

**Figure 3:**
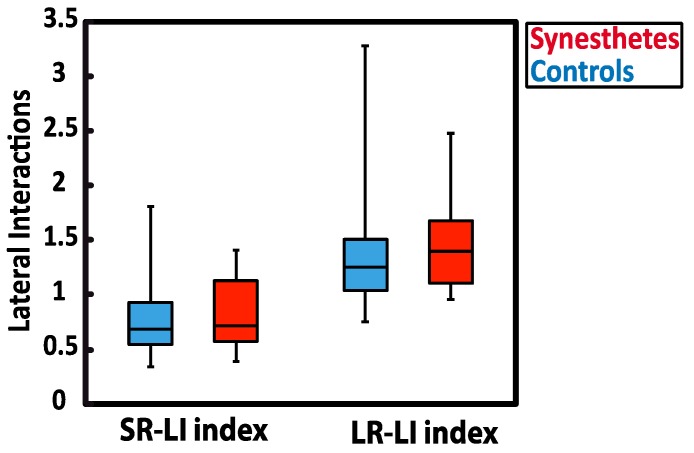
Lateral interaction indices for synesthetes (red) and controls (blue). The SR-LI index measures the relationship between F1 and F2 magnitudes of the W/D response and reflects the activity of short-range lateral interactions. The LR- LI index measures the magnitude change in F2 of the P-W/D in reference to the F2 magnitude of the W/D and reflects long-range lateral interaction activity. The whiskers indicate the minimum and maximum range of the distributions, the top and bottom of the boxes show the first and third quartiles (25th and 75th percentile), and horizontal bars inside the boxes represent the medians.

With respect to magnitude-based indices of lateral interaction activity (Figure 3), no group differences were detected. Indeed, based on magnitude responses elicited by the W/D and the P-W/D, the calculated SR-LI index (U = 54, *p* = 0.629, *r* = 0.062, exact significance) was not significantly different between the synesthetic (median = 0.714) and the control group (median = 0.686). In addition, no significant difference was obtained for the LR-LI index (U = 53, *p* = 0.589, *r* = 0.112, exact significance) between synesthetes (median = 1.251) and controls (median = 1.397). Thus, these results do not support the presence of atypical local lateral interactions (short or long-range type) in the primary visual cortex of the tested synesthetes. Higher scores were obtained for the LR-LI index in both groups, which suggests stronger recorded activity in the long-range lateral interactions.

Given the evidence in the literature suggesting that dynamic connectivity may differ between synesthetes depending on whether they perceive colors in the “outside world” (projector type) or in the “mind’s eye” (associator type) [22], we investigated whether lateral interaction indices measured in the synesthetes were associated with such individual differences.

**Figure 4:**
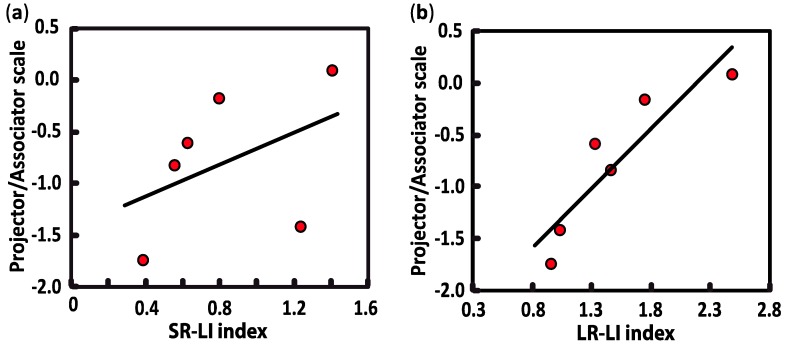
Pearson correlations between projector/associator score measured in synesthetic participants and lateral interaction indices, i.e., the SR-LI index (**a**) and the LR-LI index (**b**). Positive scores are ranked as “projector” (synesthetic colors perceived as being “outside of the body”) and negative scores are considered to be “associator” (synesthetic colors being experienced in the “mind’s eye”).

To do so, we correlated lateral interactions with the “projector/associator” profile, which was collected with the online synesthesia battery [3] and administrated to all synesthetes. The projector/associator profile was obtained from 12 questions (5-point Likert-scale) assessing each respondent’s level of agreement on a list of statements about the way synesthesia is typically experienced. Negative scores suggest that synesthetic associations are perceived to a greater extend in the “mind’s eye” (associator indicator), while positive scores reflect more of a “out of the mind” perception of synesthetic colors (projector indicator). In our study, the scores of synesthetes ranked from −1.745 to +0.085, with only one synesthete in the sample being classified as a definite projector. As illustrated in Figure 4, the SR-LI index was not significantly related to projector/associator scores (SR-LI index: *r*(6) = 0.439, *p* = 0.384). However, a positive correlation was found between projector/associator scores and the magnitude values in the LR- LI index (LR-LI index: *r*(6) = 0.907, *p* = 0.013) (Figure 3). Thus, higher ratings of projected experience on the self-rated projector/associator questionnaire was associated with a higher LR- LI index.

## 4. Discussion

In this study, we examined brain responses to visual lateral interactions in synesthetes using ssVEPs over the occipital cortex. We found that the signal of one of the components induced by the W/D stimulus presentation (the fundamental frequency; F1) was lower in synesthetes. Despite this finding, no distinguishable pattern was observed between the control and synesthetic groups when lateral interactions were assessed via magnitude-based indices. Thus, we failed to confirm the hypothesis that V1 lateral interaction processing is altered in synesthesia. As a result, it is unlikely that the primary visual cortex exhibits patterns of atypical connectivity, which characterizes the synesthetic brain [20,21,22]. Interestingly, the long-range lateral interaction index was highly correlated with the synesthetic projected/associated subjective experience, indicating a relationship between the degree to which the synesthetic experience is projected outside of the individual and the strength of long-range lateral interactions.

In our study, synesthetic responses at the fundamental frequency (F1) component in the W/D elicited lower signal coherence. However, this result did not impact measures assessing the neural activity related to lateral interactions, which were found to be comparable to the controls. In other words, the integrity of lateral interactions seems to be immune to the degree of signal coherence. Our findings are in line with those reported by Kim et al. [29] in schizophrenic participants. The authors examined lateral interactions in the visual cortex using the same W/D paradigm in a group of people suffering from schizophrenia and in a group of neurotypicals. Relative to controls, schizophrenic participants exhibited a significantly lower signal coherence of the second harmonic (not at fundamental frequency). However, the lateral interaction measures did not differ between groups, suggesting that local neuronal interactions remained unaltered. Thus, the authors interpreted their results as a potential deficit in early visual processing, without changes in local neural networks. The W/D paradigm was also studied in individuals diagnosed with autism spectrum disorder (ASD) [28]. Again, no differences in lateral interaction indices were found between the ASD and control group, in spite of the fact that ASD has previously been linked to atypical neural mechanisms, either in the visual brain connectivity or in visual perception [40,41,42]. Based on these results, one may wonder whether the W/D paradigm lacks the sensitivity to detect subtle alterations of lateral interaction processing in the visual cortex. However, this would be unlikely since lateral interaction indices have been found to change following exposure to drug treatment in epileptic patients [30], as well as between attacks in migraine patients [31].

The neural variability and noise observed in response to the W/D stimulus in synesthetes as revealed by the MSC analyses may reflect early visual processing alterations. This interpretation is consistent with previous findings that point to the presence of functional atypicality in the primary visual cortex in synesthetes, even though the nature of the contribution of V1 in synesthesia is still a matter of debate [14,15,23,24,25,26]. However, most of these studies showed increased activity in the visual cortex compared to controls. Interestingly, Barnett et al. [26] raise the possibility that some visual processes in synesthesia might be enhanced, while others may be diminished. In their study, they recorded visual evoked potentials and found consistent increases in the P1 response to high spatial frequency Gabor patches at several contrast levels (8 to 64%) and a decrease in responsiveness under the presentation of low spatial frequency Gabor patches (4% contrast).

Consistent with previous studies, we failed to detect the presence of atypical lateral interactions in the primary visual cortex, but we identified that differences in the synesthetic experience (projector vs associator) may be linked to modulations in the hard wiring of long-range lateral interactions. More specifically, our correlation analyses showed that the more the synesthetic experience is perceived as being “outside of the body” (projector synesthete), the higher the activity related to long-range lateral interactions will be. In other words, the more the synesthetic experience is perceived as being “within the mind’s eye” (associator synesthete), the weaker the activity related to long-range lateral interactions will be. Projector-type synesthesia has been linked to better perceptual performance in a visual crowding task [15], which is thought to rely on various brain processes, including lateral interactions at an early level of central visual processing [43]. Moreover, grapheme-color projector synesthetes are faster than non-synesthetes, or associator synesthetes, at recognizing masked letters [44] and color naming [8,11,15]. At the neurological level, differences in brain activity and brain structures have been reported between projector and associator synesthetes [16,22,45,46,47], including differences in the occipital area [45,46]. These studies suggest that projector synesthetes have stronger color percepts, which are associated with some patterns of functional brain activation that involve early sensory areas. However, our results must be interpreted with caution as most of the synesthetes were categorized as associator types, with only one participant identified as a projector type. In addition, most tools used to classify synesthetes into projector/associator groups, including the ones that were used in the current study, are self-reported questionnaires and are therefore subjective. It also remains unclear whether this aspect of the synesthetic experience is dichotomous or on a continuum [11].

In conclusion, the occipital activity related to lateral interactions in synesthetes do not substantially differ from those observed in controls, but the ssVEP signal coherence for the fundamental frequency was weaker in synesthetes. Further investigations are needed to verify the significance of these preliminary findings with a larger sample size and a better representation of synesthetes, in particular the projector type. However, it may be challenging since only 10% of synesthetes report synesthetic sensations as being in their external space. Finally, a more comprehensive assessment of lateral interaction processing could be achieved by creating a mixed protocol that directly compares results on a neural and behavioral measure, such as a lateral masking task [48].
